# Supplementary material for: Efficacy of rupatadine in reducing the incidence of dengue haemorrhagic fever in patients with acute dengue: A randomised, double blind, placebo-controlled trial
Source: PLoS Negl Trop Dis. 2022 Jun 1;16(6):e0010123. doi: 10.1371/journal.pntd.0010123 (PMC9191706; doi:10.1371/journal.pntd.0010123)
Supplement: S1 Appendix — (DOCX) [file pntd.0010123.s001.docx]

| **Title** | **The efficacy of rupatadine in treating dengue: a randomised, double blind, placebo controlled trial** |
| --- | --- |
| **Principal Investigator** | Prof. Neelika Malavige: Department of Microbiology, USJ |
| **Investigators** | 1. Dr. Ananda Wijewickrama: National Infectious Diseases Hospital, Sri Lanka  2. Prof. Chandanie Wanigatunga: Department of Pharmacology, USJ  3. Dr. Shamini Prathapan: Department of Community Medicine, USJ  4. Dr. Chandima Jeewandara: Department of Family Medicine, USJ  5. Dr. Harsha Dissanayake: Department of Anatomy, USJ  6. Dr. Samitha Fernando: Centre for Dengue Research, USJ  7. Mr, Laksiri Gomes: Centre for Dengue Research, USJ  8. Dr. Pathum Perera: Centre for Dengue Research, USJ  9. Geethal Bandara: Centre for Dengue Research, USJ  10. Graham Ogg: MRC Human Immunology Unit, University of Oxford |
| **Sponsor**: | University of Sri Jayewardenapura |
| **Funder:** | Centre for Dengue Research, University of Sri Jayewardenapura |

**Confidentiality Statement**

This document contains confidential information that must not be disclosed to anyone other than the Sponsor, the Investigator Team, SCOCT, host organisation, SLMA Clinical trial registry and members of the Research Ethics Committee, unless authorised to do so.

TABLE OF CONTENTS

[1 KEY TRIAL CONTACTS 4](#_Toc480915605)

[2 SYNOPSIS 5](#_Toc480915606)

[3 ABBREVIATIONS 7](#_Toc480915607)

[4 BACKGROUND AND RATIONALE 8](#_Toc480915608)

[5 OBJECTIVES AND OUTCOME MEASURES 14](#_Toc480915609)

[Primary Study Endpoints 14](#_Toc480915610)

[6 TRIAL DESIGN 16](#_Toc480915611)

[7 PARTICIPANT IDENTIFICATION 19](#_Toc480915612)

[8 TRIAL PROCEDURES 21](#_Toc480915613)

[8.1 Recruitment: 21](#_Toc480915614)

[8.2 Screening and Eligibility Assessment 21](#_Toc480915615)

[8.3 Informed Consent 21](#_Toc480915616)

[8.4 Randomisation, blinding and code-breaking 22](#_Toc480915617)

[8.5 Base line assessment 23](#_Toc480915618)

[8.6 Subsequent Visits 23](#_Toc480915619)

[9 INVESTIGATIONAL MEDICINAL PRODUCT (IMP) 24](#_Toc480915620)

[9.1 IMP Description 24](#_Toc480915621)

[9.2 Storage of IMP 25](#_Toc480915622)

[9.4 Accountability of the Trial Treatment 25](#_Toc480915623)

[9.5 Concomitant Medication 25](#_Toc480915624)

[9.6 Post-trial Treatment 25](#_Toc480915625)

[10 SAFETY REPORTING 26](#_Toc480915626)

[10.1 Definitions 26](#_Toc480915627)

[10.2 Causality 27](#_Toc480915628)

[10.3 Procedures for Recording Adverse Events 28](#_Toc480915629)

[10.4 Reporting Procedures for Serious Adverse Events 28](#_Toc480915630)

[10.5 SUSAR Reporting 28](#_Toc480915631)

[10.6 Data Safety Monitoring Committee 28](#_Toc480915632)

[11 STATISTICS 29](#_Toc480915633)

[11.1 Description of Statistical Methods 29](#_Toc480915634)

[11.2 The Number of Participants 30](#_Toc480915635)

[11.3 The Level of Statistical Significance 30](#_Toc480915636)

[11.4 Criteria for the Termination of the Trial 30](#_Toc480915637)

[11.5 Procedure for Accounting for Missing, Unused, and Spurious Data. 30](#_Toc480915638)

[11.6 Inclusion in Analysis 31](#_Toc480915639)

[11.7 Procedures for Reporting any Deviation(s) from the Original Statistical Plan 31](#_Toc480915640)

[12 DATA MANAGEMENT 31](#_Toc480915641)

[12.1 Source Data 31](#_Toc480915642)

[12.2 Access to Data 31](#_Toc480915643)

[12.3 Data Recording and Record Keeping 31](#_Toc480915644)

[13 QUALITY ASSURANCE PROCEDURES 32](#_Toc480915645)

[14 SERIOUS BREACHES 32](#_Toc480915646)

[15 ETHICAL AND REGULATORY CONSIDERATIONS 32](#_Toc480915647)

[15.1 Declaration of Helsinki 32](#_Toc480915648)

[15.2 Approvals 33](#_Toc480915649)

[15.3 Reporting 33](#_Toc480915650)

[15.4 Participant Confidentiality 33](#_Toc480915651)

[15.5 Expenses and Benefits 33](#_Toc480915652)

[16 FINANCE AND INSURANCE 34](#_Toc480915653)

[17 PUBLICATION POLICY 34](#_Toc480915654)

[18 REFERENCES 34](#_Toc480915655)

# KEY TRIAL CONTACTS

| **Chief Investigator** | Prof. Neelika Malavige, Centre for Dengue Research, Faculty of Medical Sciences, University of Sri Jayewardenapura.  Email: [neelika@sjp.ac.lk](mailto:neelika@sjp.ac.lk)  Phone: 0772443193 |
| --- | --- |
| **Sponsor** | Centre for Dengue Research, Faculty of Medical Sciences, University of Sri Jayewardenapura.  Email:  Phone: |
| **Statistician** | Dr. Shamini Prathapan: Department of Community Medicine, Faculty of Medical Sciences, University of Sri Jayewardenapura.  Email: [Shamini@sjp.ac.lk](mailto:Shamini@sjp.ac.lk)  Phone: 0714852269 |
| **Committees** | Prof. Renu Wickramasinghe: Chairperson, Ethics Review Committee, Faculty of Medical Sciences, University of Sri Jayewardenapura  Email: [renuwick@gmail.com](mailto:renuwick@gmail.com)  Phone: 0777571821 |

# SYNOPSIS

| Trial Title | The efficacy of rupatadine in treating dengue: a randomised, double blinded, placebo controlled trial | |
| --- | --- | --- |
| Short title | Efficacy of rupatadine in dengue | |
| Clinical Phase | Phase 2 | |
| Trial Design | Randomised, double blinded, placebo controlled | |
| Trial Participants | Patients with dengue infection | |
| Planned Sample Size | 280 with 140 each in each study arm | |
| Treatment duration | 5 days | |
| Follow up duration | 30 days of illness | |
| Planned Trial Period | 1 year | |
|  | Objectives | Outcome Measures |
| Primary | To determine the proportions of dengue patients who develop DHF when treated with and without rupatadine | Therefore, the reduction of the proportion of individuals who develop fluid leakage (DHF) will be assessed.  Evidence of plasma leakage: evidence of plasma leakage considered as detection of free fluid in the abdomen or by the presence of a pleural effusion by USScan and/or by the presence of a rise in haematocrit of >20% of the baseline. |
| Secondary | Investigate whether rupatadine treatment leads to a. reduction in complications such as liver failure.  b. Reduction in development of shock:  c. Need of colloids:  d. Need of blood transfusion/s:  e. Duration of the illness.  f. Sleep disturbance | Liver function tests, blood clotting, colloid infusion volumes, blood transfusion volumes, length of acute illness, sleep scores. |
| Investigational Medicinal Product(s) | Rupatadine 40mg | |
| Formulation, Dose, Route of Administration | Four 10mg tablets of rupatadine to be given orally, and placebo | |

# ABBREVIATIONS

| AE | Adverse event |
| --- | --- |
| AR | Adverse reaction |
| CDR | Centre for Dengue Research |
| CRF | Case Report Form |
| CT | Clinical Trials |
| DF | Dengue Fever |
| DHF | Dengue Haemorrhagic fever |
| DSMB | Data Safety Monitoring Board |
| ERC | Ethic Review Committee |
| GCP | Good Clinical Practice |
| GP | General Practitioner |
| ICF | Informed Consent Form |
| ICH | International Conference on Harmonisation |
| OPD | Out patients department |
| PI | Principal Investigator |
| PIL | Participant/ Patient Information Leaflet |
| SAE | Serious Adverse Event |
| SAR | Serious Adverse Reaction |
| SCOCT | Sub Committee on Clinical Trials |
| SLCTR | Sri Lanka Clinical Trial Registry |
| SMPC | Summary of Medicinal Product Characteristics |
| SUSAR | Suspected Unexpected Serious Adverse Reactions |
| USJ | University of Sri Jayewardenapura |

# BACKGROUND AND RATIONALE

Dengue viral infections represent one of the most rapidly emerging mosquito borne infections in the world, spreading to many geographical regions and causing almost 100 million apparent dengue infections each year (1). From 2005 to 2015, although the mortality rates due to many infectious diseases decreased, the deaths due to dengue increased by 48.7%, resulting in an estimated 18,400 deaths in 2015 (2). Although there is now a dengue vaccine, which is licenced to be used in individuals over 9 years of age in several countries, it is only recommended in countries with high rates of dengue seroprevalence, due to its varied efficacy (3). Intense monitoring with meticulous fluid control is currently the only option in the management of acute dengue infection, as specific treatments for dengue are not yet available.

Severe dengue is characterized by clinically detectable vascular leak, which can lead to haemodynamic instability and shock. Vascular leakage is a hallmark of dengue haemorrhagic fever (DHF) and is thought to occur due to endothelial dysfunction(4) resulting in increased vascular permeability. We recently reported that platelet activating factor (PAF) is elevated during acute dengue infection and is an important mediator of vascular leak (5). Therefore, we used rupatadine in a phase 2 trial to determine its efficacy in treating acute dengue, as it had PAF receptor blocking activities. The clinical trial was a phase II, randomized controlled study, initially consisting of three arms, which were rupatadine 10mg, rupatadine 40mg and placebo. This study was carried out at a dedicated dengue management unit in a tertiary care hospital in Colombo District, Sri Lanka (Infections Diseases Hospital) in 2015/6. Patients who had clinical features suggestive of an acute dengue infection, with a duration of illness ≤ 5 days, and who did not show any evidence of vascular leak and also tested positive for dengue NS1 antigen were recruited. An interim analysis was carried out after recruiting half of the number of patients (n=120). At the interim analysis, it was found that 40mg rupatadine was safe in acute dengue, and that 10mg of rupatadine did not seem to show any benefit when compared to the placebo, and so the rupatadine 10mg arm was discontinued.

The number of adverse events (AE) were similar in those on rupatadine 40mg and on the placebo and all AE completely resolved (Table 1). Two patients (2.98%) in the placebo group developed a SAE, namely acute liver failure. AE such as hepatitis (ALT>250U/L) was more frequent in those on the placebo as 6 (8.9%) patients developed hepatitis when compared to 3 (4.5%) on rupatadine 40mg. Severe thrombocytopenia (<5×10^9^/L) was seen in one patient in the placebo group. However, thromobocyotopenia and hepatitis are known features of dengue and therefore, unlikely to be due to drugs.

|  | Rupatadine 40mg  N=66 | Placebo  N=67 |
| --- | --- | --- |
| Abdominal pain (%) | 17 (25.7) | 21 (31.3) |
| Vomiting (%) | 21 (31.8) | 21 (31.3) |
| Diarrhoea (%) | 27 (40.9) | 24 (35.8) |
| Hepatitis (%) (ALT>250/L) | 3 (4.5) | 6 (8.9) |
| Low platelet counts  <20 ×10^9^/L (%)  20 to 50×10^9^/L (%) | 12 (18.2)  17 (25.7) | 14 (20.9)  21 (31.3) |
| High ALT (>200 U/L) (%) | 9 (13.6) | 12 (17.9) |
| High AST (>200 U/L) (%) | 14 (21.2) | 18 (26.9) |
| Low white cell count  (<1×10^9^/L) (%) | 0 (0) | 0 (0) |

**Table 1: Adverse events experienced by patients on rupatadine 40mg and placebo**

Although, the overall proportion of patients who developed pleural effusion and ascites was similar in both arms, the mean maximum height of the pleural effusion, was lower throughout the illness in those who were on rupatadine when compared to those on the placebo. We did find a >50% reduction in the mean of the maximum height of the pleural effusion, which was seen on day 8 of the illness in the rupatadine arm (1.7, SD±0.4 cm) when compared to the placebo arm (2.9, SD±1.3 cm). The AST on day 7 of illness was significantly less (p<0.05) in those who were on rupaadine compared to placebo; and rupatadine mildly but significantly reduced thrombocytopenia during the study (Fig 1).


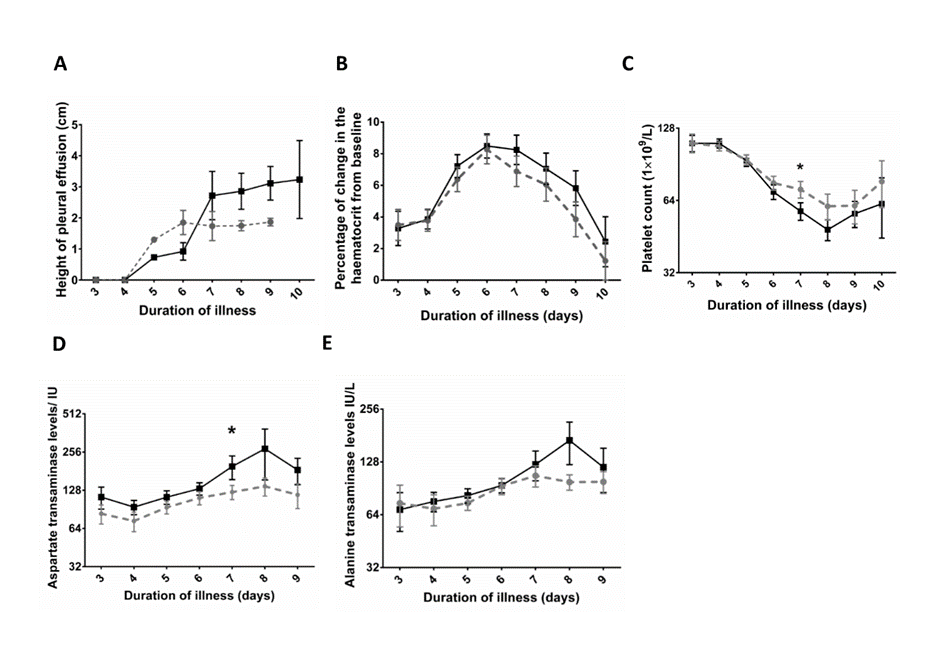


**Fig 1:** **Changes in clinical and laboratory parameters in patients on 40mg of rupatadine and placebo**. (A) Height of the pleural effusion was measured by daily ultrasound scan and expressed in time (days) since onset of symptoms. Dotted line represents patients receiving rupatadine (40mg daily, n=66), and solid lines represent those receiving placebo (n=67). (B) Haematocrit was measured daily and expressed as percentage change from the baseline value at presentation. Serum aspartate transaminase levels (C), alanine transaminase (D), and blood platelet counts were measured (E). The bars represent mean and SEM. *P<0.05.

6 (8.9%) patients on the placebo developed significant bleeding, which was considered as any bleeding that was not restricted to the skin. Four of these participants reported per vaginal bleeding (in 3 patients lasting for >3 days), one reported melena and one developed epistaxis. In contrast, 2 (3%) of patients in the rupatadine group developed per vaginal bleeding, which did not last for more than one day. There were no differences in other clinical and laboratory parameters, or the duration of illness in those on the placebo and rupatadine. However, there was a trend for an effect of rupatadine on reducing the overall rise in haematocrits and the rise in ALT.

*Early efficacy of rupatadine*

17 (25.7%) who were assigned to the rupatadine arm and 21 (31.3%) assigned to the placebo arm were recruited at ≤3 days of illness. The overall duration of illness was significantly less in the early treatment group (p=0.03). Of those who were on rupatadine and treated, only one patient (5.9%), developed a pleural effusion, when compared to 6 (12.2%) of those who were recruited later than 3 days (12.2%) (Table 2), but this did not reach significance. Although the proportion of patients who developed ascites was similar, the severity was less in the early treated group in which only one patient (5.9%) developed moderate ascites compared to nine (18.4%) of those who were recruited late. Of those who were recruited early only one (5.9%) patient was given colloids (dextran) in anticipation of subsequent deterioration, when compared to 5 (10.2%) of those who were recruited later. Only one patient (5.9%) had a rise in had the haematocrit rise >20% at some time during the when compared to 8 (16.3%) in the late treatment group.

|  | **Placebo**  **Recruited ≤ 3 days**  **N=21** | **Rupatadine**  **Recruited ≤3 days**  **N=17** | **P value** |
| --- | --- | --- | --- |
| Pleural effusion by Ultra Sound Scan | 3 (14.3) | 1 (5.9) | NA |
| Ascites by Ultra sound scan | 9 (42.8) | 4 (23.5) | 0.3 |
| Extent of Ascites as measured by US Scan  Mild  Moderate | 5 (23.8)  4 (19.4) | 3 (17.6)  1 (5.9) | NA |
| Duration of illness  Median (IQR)  Mean (SD±) | 7 (6 to 8.5)  7.1 (1.8) | 7 (5.5 to 7)  6.3 (1.5) | 0.2 |
| Haematocrit >20% rise from baseline | 4 (19.4) | 1 (5.9) | 0.3 |
| Low platelet counts  <20,000  20,00 to 50,000 | 5 (23.8 )  7 (33.3) | 2 (11.7)  7 (41.2) | NA |
| High ALT (>200 U/L) | 2 (9.5) | 1 (5.9) | NA |
| High AST (>200 U/L) | 6 (28.5) | 1 (5.9) | NA |

**Table 2: Clinical and laboratory features of patients who were on rupatadine on the placebo who were recruited on ≤ 3 days**

Those who were in the placebo arm were more likely to have pleural effusions and ascites, more likely to have their haematocrits rise >20%, more likely to have needed dextran and more likely to have AST levels of >200 U/L. When rupatadine was given earlier, one of the primary end points was met, namely a >50% or more reduction in the fluid leakage, as quantity of fluid leakage as assessed by the degree of ascites (mild or moderate). Furthermore, there was a reduction in the proportion of patients who developed pleural effusions (12.2% late vs 5.9% early), moderate ascites (18.4% vs 5.9%) and a >20% rise in the haematocrit (16.3% vs 5.9%). There were no differences in the clinical outcomes and the duration of illness in those who were recruited early (≤ 3 days of illness) when compared to those who were recruited late to the placebo arm of the study. Although those who were recruited early to the placebo arm were less likely to have ALT levels of >200 U/L, the proportion of patients with AST levels >200 U/L were similar in both groups.

**Safety of rupatadine**

In the phase 2 randomized, placebo controlled trial, 40mg of rupatadine was found to be safe and the number of adverse events (AE) were similar in those on rupatadine 40mg and on the placebo and all AE completely resolved. However, the adverse effects on the liver such as development of hepatitis was less in those who were on rupatadine 40mg, when compared to the placebo. In addition, bleeding tendencies were also less. Since both hepatitis and bleeding manifestations are known features of dengue, these clinical features are unlikely to be due to the drugs.

Rupatadine is an orally available second generation antihistamine known to have long acting dual histamine 1 receptor blocking activities and PAF receptor blocking activities for treatment of allergic disease and chronic urticaria (6, 7). Rupatadine has been shown to be well tolerated in many clinical trials in patients who were treated for allergic rhinitis (8, 9), and off-label doses of up to 40mg/day have shown to be well tolerated in chronic urticaria (10). Rupatadine has not shown cardiac adverse effects even at administration of 100mg in healthy individuals (11) (12). It was shown to have less typical side effects of earlier antihistamines such as somnolence, fatigue, headache, impaired memory and learning and sedation (6). A study which determined the effect of rupatadine on those on lorazepam showed that repeated doses of rupatadine had no effect on psychomotor impairment or subjective performance when compared to a placebo (13).

**Justification**

Dengue infections cause significant mortality and morbidity in Sri Lanka and in many tropical and subtropical countries. The estimated annual global cost associated with dengue is $8.9 billion (14). Intense monitoring with meticulous fluid control is currently the only option in the management of acute dengue infection, as specific treatments for dengue are not yet available. There is clearly a major unmet clinical need for a safe effective therapy that can be realistically used in a practical way in resource-poor communities where the bulk of infections occur. In our recently concluded randomised, placebo controlled, phase 2 trial, we found that rupatadine 40mg was safe and well tolerated in patients with acute dengue, and it reduced fluid leakage and other complications associated with dengue, particularly when given early (≤ 3 days from symptom onset) in clinical disease. Therefore, as the first potential active intervention in the treatment of dengue infection, we believe that the efficacy of rupatadine 40mg should be further evaluated by trials conducted in the outpatient setting.

# OBJECTIVES AND OUTCOME MEASURES

**General objective**

To assess the of 40mg of rupatadine on reduction of incidence of DHF and its associated complications in those with dengue viral infections

**Specific objectives**

To assess the effect of 40 mg of rupatadine in reduction of the incidence of DHF

- To determine the effect of 40 mg of rupatadine on reduction of incidence of complications associated with DHF such as acute liver failure, hepatitis, the need for colloids, significant bleeding manifestations
- To determine if rupatadine reduces the duration of post illness fatigue and sleep disturbances

## Primary Study Endpoints

Reduction in proportion of individuals who are treated with rupatadine who develop DHF (fluid leakage). In our earlier phase 2 study, we found that approximately 25% of hospitalized patients with dengue infection develop fluid leakage when assessed by serial US Scanning. Therefore, the reduction of the proportion of individuals who develop fluid leakage (DHF) will be assessed. As this trial is conducted in an OPD setting, those who are not admitted will considered to only have DF as any patient who is suspected to develop DHF (fluid leakage) will be admitted. By the time of discharge from hospital, as the patient would have recovered at this time point, if the patient had not developed fluid leakage, he/she will considered as not had DHF.

Evidence of plasma leakage will be considered as:

- detection of free fluid in the abdomen or by the presence of a pleural effusion by USScan
- Or by the presence of a rise in haematocrit of >20% of the baseline.

The haematocrit of the patients will be monitored at least three times a day. If the haematocrit at any given time point is >20% of the patients baseline the patient will be considered as having fluid leakage according to the Sri Lanka National Dengue Management guidelines (15). The patients will also be monitored by daily serial ultrasound scans done by the same person using a portable USScanner in the ward. The scans will be recorded and reassessed by a consultant radiologist to ensure accuracy. Presence of fluid in the pleural and peritoneal cavities or a rise in haematocrit of >20% will be considered as evidence of plasma leakage.

Ultrasound scanning will be done in the supine position to assess the peritoneal fluid: in the hepatorenal angle, subdiaphragmatic position, in paracolic gutters and in the pelvis, and patient will be kept seated upright on the examination bed for 2 minutes for all the pleural fluid to gravitate in to costodiaphragmatic recesses and scanned from the back of the chest hands raised up and crossed back of the head, mid and posterior axillary lines and in seated up position.

**Secondary end point**

a. Reduction in complications such as liver failure with use of rupatadine. Liver failure will be diagnosed based on the following criteria. All of the 3 following criteria should be present (16).

- Absence of any previously known liver disease
- A prolonged prothrombin time of 4-6 seconds or more
- A INR of >1.5

b. Reduction in development of shock: Shock will be defined as a pulse pressure of ≤20 mmHg or a drop in the systolic blood pressure of 30mmHg or more

c. Reduction in need of colloids: use of dextran will be considered as the patient having significant fluid leakage as per National Guidelines

d. Reduction in need of blood transfusion/s: as per National Guidelines. Use of even 1 blood transfusion will be considered as the patient having significant fluid leakage

e. Reduction in duration of the illness. The first day of the illness will be defined as the day in which the patient developed fever. The day of recovery will be defined when **all** of the following 3 criteria are fulfilled:

- Patient afebrile for 24 hours
- Platelet counts of >50,000 cells/mm^3^ or a rise of 20% from the lowest value
- Return of the haematocrit to the patients baseline

f. Reduction in sleep disturbances: a reduction in sleep disturbances will be assessed by an eight point scoring system, known as the sleep condition indicator (17). This score assessed, difficulty in falling asleep, waking up in the night, quality of sleep, any effect of poor sleep in concentration, relationships, productivity and general wellbeing. The maximum score attainable is 32 and higher scores indicate better sleep quality.

# TRIAL DESIGN

This study is a randomized, double blinded, placebo controlled trial. The efficacy of the drug will be measured by how well it will be able to it reduce the proportion of patients who develop DHF. As secondary endpoints we hope to evaluate if it reduces the proportion of patients requiring hospital admission and also complications associated with dengue.

**Selection of patients**: Patients, who are suspected to be having a dengue infection, will be tested for dengue NS1 antigen test. These tests will be done free of charge for all patients with suspected dengue infection at the OPDs of the respective hospital. The patients who are confirmed to be having acute dengue infection and who fulfil the inclusion criteria and who do not have the exclusion criteria will be approached by a member of the team of investigators or by a medical officer of the unit who will be specially trained in obtaining informed written consent.

At the initial visit they will be assessed for the presence of dengue warning signs, their blood pressure and pulse rate recorded and a base line FBC done. They will be advised to come to the OPD for daily assessments and will be asked to come sooner if they develop persistent vomiting, feeling faint or develop any bleeding manifestations. They will be asked not to take any other over the counter drugs apart from any drugs given from the hospital (paracetamol) as per standard management of dengue.

**Data collection:** An interviewer administered questionnaire will be used to record basic demographic details of the patient. In those who are only assessed in the OPD setting (those who do not require hospital admission), basic demographic details, any co-morbid illnesses (diabetes, hypertension, asthma and allergic rhinitis), clinical features along with the daily full blood counts (FBCs) will be recorded liver function tests (Annexure 1). If the patient is admitted to hospital, additional clinical data such as hepatomegaly, presence or absence of serous effusion and additional investigations such as liver function tests, which are routinely done as standard management of dengue will be recorded (Annexure 2).

Interviews carried out on group of 15 patients revealed that reduction of fever, headache, body aches and return of appetite were important parameters for them. In addition, absence of post illness sleep disturbances and fatigue were also listed as important parameters by the patients. Therefore, we will record and measure these parameters in the daily assessments and also during follow up visits and telephone interviews.

A serial number will be used to identify the patient and the patient data will be entered in a data base where only the patient serial number will be used. Disease severity will be classified according to the 2011 WHO criteria (15).

**Patient follow up**

All patients will be closely monitored during the trial as they will have to visit the study site (OPD) daily for assessment and for blood tests (FBC) as standard management of patients with dengue infection. 4ml of blood will be taken at the OPD for FBCs which are done at the hospital (NIID) as standard management of patients with dengue. All patients will attend daily, and assessed for presence of dengue warning signs and the blood pressure and pulse rate will be recorded. During the visits we will find out the day the patient recovers from illness as defined by the following criteria.

Day of recovery is defined when **all three** criteria below are fulfilled:

-Patient afebrile for 24 hours

-Platelet counts of >50,000 cells/mm^3^ or a rise of 20% from the lowest value

-Return of the haematocrit to the patient’s baselineIf the patient need to attend any OPD visits after the day of recovery, they will be paid Rs. 500 as reimbursement for travel. In each visit the patients will be assessed and monitored in an identical manner.

They will be asked to come 14 days after the onset of illness and the patients general wellbeing will be assessed by patient reported outcome measures including presence of any sleep disturbances (Annexure 3). At the 14 day follow up visit, a blood sample will be taken (4ml) and will be assessed for the presence of dengue IgM and IgG specific antibodies at the Centre for Dengue Research (CDR), University of Sri Jayewardenapura.

The patient will also be contacted 30 days after the onset of illness, by phone to assess the general wellbeing and presence of any sleep disturbances. At the follow up an interviewer administered questionnaire will be used to find out the post viral fatigue and sleep disturbances as previously used in similar studies (17-19). The modified fatigue scale has been previously used to evaluate post viral fatigue following West Nile virus infection (19), which is a viral infection similar to dengue. A fatigue questionnaire has been previously used to evaluate fatigue following dengue infection(18). Before administering these questionnaires to patients in this study (annexure 1), we will validate both questionnaires in this country by translating both to Sinhalese and Tamil.

**Blood samples**: 4ml of blood will be obtained at the time of recruitment when the patient is being bled as per routine management of patients with dengue infection and again 14 days later to determine if the patient has primary or secondary dengue. Dengue NS1 will be performed at the OPD itself for with the initial blood sample and dengue virus serotype will be done at the CDR. The antibody testing will be done with the second blood sample at the CDR.

**Dengue virus detection assays**

1. Dengue viral loads will be determined by quantitative real time PCR in all of the patients allocated to each arm of the study. These will be done at the Centre for Dengue Research, USJ.

2. ELISA for dengue antibodies: We will be doing ELISA for dengue IgM and IgG antibodies to determine if the patient is having a primary or a secondary dengue infection on the second sample of blood obtained. As the immune responses are quite different in primary and secondary dengue infections we hope to see if the effects of rupatadine are different in these groups of patients. This ELISA assay has been validated as both sensitive and specific for primary and secondary dengue virus infections (20, 21). The combined use of both IgM and IgG been shown to have a sensitivity of 99% and a specificity of 96% to distinguish between primary and secondary dengue infections(21). Patients who only have dengue virus specific IgM were classified as having a primary dengue (PD) infection while those who had a positive result for both IgM and IgG will be classified as having a secondary dengue (SD infection) (22).

# PARTICIPANT IDENTIFICATION

**Patient recruitment:** Patients with a suspected dengue infection, between the ages of 18 to 60, will be recruited from the OPDs of the NIID following informed written consent.

**Inclusion criteria**: All patients with confirmed dengue infection by a positive dengue NS1 antigen detection test who give informed written consent and who fulfil **all** of the following criteria will be recruited.

- **Age:** between 18-60
- **Duration of illness**: <3 days and who do not show any evidence of vascular leak. This is because in our earlier phase 2 trial, we found that the complications and the reduction in proportion of patients developing DHF was less when given early (3 ≤ days).
- **Evidence of dengue infection:** Dengue NS1 antigen test will be done in these patients and only those who are dengue antigen positive will be included. Although the sensitivity of the NS1 is less in those who are infected with the DENV-2 serotype, and even though those who are NS1 negative can also be having dengue, we will only include those who have are NS1 positive, as we believe it is important to have some evidence that the patient is infected with the dengue virus at the time of recruitment. The virus serotype and the viral loads will be done in all patients at the Centre for Dengue Research (please see below for details).

The dengue NS1 antigen will be done by rapid immununochromatographic assays (rapid strip tests) at the OPD.

**Exclusion Criteria**

- Those who already have fluid leakage
- Fever for >3 days
- pregnant women
- those who have known allergies to antihistamines
- Individuals who are homeless, who are dependent on alcohol dependence or abuse drugs as it will be difficult to monitor these patients in the community
- Those who are unable to take the drugs orally
- Those who have known hepatic impairment defined as: All of the 3 following criteria should be present (16). Patients will be inquired about pre-existing liver disease and their clinical records accessed to find out if they have any of the following.
- Absence of any previously known liver disease
- A prolonged prothromin time of 4-6 seconds or more
- A INR of >1.5
- Those with known renal impairment will be defined as those who fulfill either of the following criteria regardless of age, hypertension and diabetes (23). Patients will be enquired regarding presence of diabetes and hypertension and will be enquired regarding any pre-existing renal disease (annexure 1). Their clinical records will be accessed to determine if they have any of the following.

- predicted GFR <60 ml/min per 1.73 m^2^

- Hereditary kidney disease

- Recurrent or extensive nephrolithiasis

Those with other pre-existing medical conditions that the PI considers may impact on interpretation of the study.

# TRIAL PROCEDURES

## 8.1 Recruitment:

Patients, who are suspected to be having a dengue infection, who fulfil the inclusion criteria and who do not have the exclusion criteria will be approached by a member of the team of investigators or by a medical officer of the unit who will be specially trained in obtaining proper consent.

Once consent is given, they will be tested for dengue NS1 antigen test. These tests will be done free of charge for all patients with suspected dengue infection at the OPD of the hospital. The patients who are confirmed to be having acute dengue infection and they will be recruited to the study.

## 8.2 Screening and Eligibility Assessment

At the initial visit they will be assessed for the presence of dengue warning signs, their blood pressure and pulse rate recorded and a base line FBC done. If the patient is found to have evidence of fluid leakage or fulfil any of the exclusion criteria in the assessment, he/she will not be recruited.

## 8.3 Informed Consent

Consent will be taken by one of the investigators in the team, who have completed GCP training. The patients will be given the patient information sheets of the study and will be given an opportunity to discuss their concerns/questions with a member of the team of investigators. Once the patient is satisfied with the information given to him/her, he/she will be given the opportunity to join the study or to decline. They will be made aware that their decision will in no way affect their other treatment, and that they are free to withdraw at any time.

## 8.4 Randomisation, blinding and code-breaking

The randomization schedule will be designed by the statistician and she will be responsible for providing the randomization codes. The tablets of the placebo and the rupatadine will be packed into envelops, which is to be given to each patient. Each envelop will contain tablets needed for the whole duration of treatment (5 days). Once the codes are provided, the packaging will be by individuals who are not involved in the study. The packed and sealed envelopes will be stored at the Centre for Dengue Research and released in small batches to be given to patients. A person who is not involved in the study will be responsible for dispersing these envelops. Therefore, all investigators and the patients will be blinded.

Once the patients are enrolled to the study a serial number is assigned in a chronological order for data entering purposes. Total number of patients will be 262 and all will be treated according to the current Dengue guidelines published by the Ministry of Health, Sri Lanka. The study groups will receive 40 mg of rupatadine in addition to the treatment guidelines (please see below). The two treatments would be named A (40mg rupatadine) and B (placebo). The patients will receive either 4 tablets of rupatadine daily for 5 days or 4 tablets of the placebo for 5 days, which will be prepacked in an envelope containing the code number. This code number will be entered in the data collection sheet along with the patient’s serial number.

Randomization will be done by using a random number table (1-10). Using this table an arbitrary point would be chosen, and the patient would be allocated according to the random number. If the number selected from the random table is between 1 to 5, the patient would be assigned to the treatment arm A. If the number selected from the Random table is between 6 to 10. The assignment information will be kept under lock and key. Neither data collectors nor the patients will be made aware of the randomization at any given point of time.

The codes will be kept with the PI and in the event of a SAE or a SAR the codes will be broken. The codes can be accessed all 24 hours. However, since we have already carried out a phase 2 trial to determine the safety of rupatadine 40mg, and was found to be safe, we do not expect any SAE. In addition, rupatadine 40mg has been used in many countries for the treatment of allergic rhinitis and chronic urticaria and SAR or SAE have not been reported.

## 8.5 Base line assessment

At the initial visit a detailed clinical history will be taken regarding the clinical features and basic demographic details such as age and gender will be recorded. A details medical history regarding past illnesses such as presence of diabetes, hypertension, hyperlipidaemia, asthma and other allergic diseases along with existing liver and kidney failure will be assessed. Those who have existing liver disease or renal disease will be excluded as defined in exclusion criteria. A detailed medical examination will be done and the temperature, heart rate, blood pressure, presence of hepatomegaly or pleural effusions or ascites will be recorded. After examination they will have a FBC done at the hospital and they will be sent home once they are assessed along with the report. They will be advised to come daily to the OPD for assessment and will be educated to seek admission earlier if they develop persistent vomiting, any bleeding manifestations or feel dizzy.

## 8.6 Subsequent Visits

Once the patients are recruited they will be asked to visit the OPD daily for assessment as per National Dengue Management guidelines. During these visits, a detailed medical history regaring vomiting, appetite, how they feel (patient based score), presence of abdominal pain will be recorded. A detailed medical examination will be done and the temperature, heart rate, blood pressure, presence of hepatomegaly or pleural effusions or ascites will be recorded. After examination they will have a FBC done at the hospital and they will be sent home once they are assessed along with the report.

Each patient will be asked to come for 5 visits during acute illness and again 14 days after the onset of illness. The patients will be contacted by phone, 30 days after the onset of illness and a questionnaire will be administered (see follow up described above for details).

**8.7 Discontinuation/withdrawal of participants from the trial**

If a patient takes more than 4 hours to recover from an episode of shock or if a patient goes in to shock for a second time during the illness (after fully recovering from the first episode of shock) we will take him/her out of the trial. Shock is defined as a pulse pressure of ≤20 mmHg or a drop in the systolic blood pressure of 30mmHg or more. Recovery of shock will be defined as an increase in the pulse pressure of >20mmHg or return of the systolic blood pressure to >30mmHg of the patients normal systolic blood pressure. All data obtained prior to that point will be included within the analysis.

**8.8 Definition of end of trial**

The trial will end at the last follow up day of the last participant.

# INVESTIGATIONAL MEDICINAL PRODUCT (IMP)

## IMP Description

- The drug will appear as a 10mg tablet
- Patients in the test groups will be given a daily dose of a four 10mg tablets (40mg) orally starting on the day of recruitment for 5 days in addition to the standard treatment as per national guidelines. The control group will receive 4 tablets of the placebo similar to rupatadine for 5 days. Rupatadine will be imported for the study purpose from Dr. Reddy India after approval from SCOCT and NMRA. The placebo will be manufactured by SPMC. The drug and the placebo will be repacked in ensure 5 days of treatment to the study participants.
- As the drug can only be administered orally it will only be given to patients who are able to take drugs orally. At any time point during the study the patient becomes ill and cannot take the drug orally the patient will be taken out of the study. All data collected until the point of withdrawal or until the point of inability to take oral medication will be used in the analysis.
- The placebo will be provided by the State Pharmaceutical Manufacturing Company. The placebo will be identical size and shape to the rupatadine tablet and will not contain ant active ingredients.

## Storage of IMP

The trial drugs (rupatadine and the placebo) will be stored in prepacked envelopes in a designated cupboard at the Centre for Dengue Research.

**9.3 Compliance with Trial Treatment**

The nursing officer who offers the drug to the patient will observe the patient taking the first dose of the drug. The patients will be asked to take the subsequent doses at home. At each daily visit, the participants will be asked if they took the drug. If the patient wishes not to take the drug at any time point in the study he/she will be taken out of the study.

## 9.4 Accountability of the Trial Treatment

Each envelope containing either the placebo or the drug will be marked by a specific number (please see above for randomization and masking). Once a particular envelope is dispersed, the date and serial number it was given to will be recorded.

## 9.5 Concomitant Medication

- Any patients on regular treatment for diseases such as hypertension, diabetes, asthma or any other chronic illness will be asked to take the medication as usual
- They will also take any other medicines such as paracetamol prescribed by the treating physicians as per National Guidelines
- Over the counter medications will not be permitted.

## 9.6 Post-trial Treatment

There will not be any provision to continue use of the drug once the trial ends.

# SAFETY REPORTING

## 10.1 Definitions

| Adverse Event (AE) | Any untoward medical occurrence in a participant to whom a medicinal product has been administered, including occurrences which are not necessarily caused by or related to that product. |
| --- | --- |
| Adverse Reaction (AR) | An untoward and unintended response in a participant to an investigational medicinal product which is related to any dose administered to that participant.  The phrase "response to an investigational medicinal product" means that a causal relationship between a trial medication and an AE is at least a reasonable possibility, i.e. the relationship cannot be ruled out.  All cases judged by either the reporting medically qualified professional or the Sponsor as having a reasonable suspected causal relationship to the trial medication qualify as adverse reactions. |
| Serious Adverse Event (SAE) | A serious adverse event is any untoward medical occurrence that:   - results in death - is life-threatening - requires inpatient hospitalisation or prolongation of existing hospitalisation - results in persistent or significant disability/incapacity - consists of a congenital anomaly or birth defect.   Other ‘important medical events’ may also be considered serious if they jeopardise the participant or require an intervention to prevent one of the above consequences.  NOTE: The term "life-threatening" in the definition of "serious" refers to an event in which the participant was at risk of death at the time of the event; it does not refer to an event which hypothetically might have caused death if it were more severe. |
| Serious Adverse Reaction (SAR) | An adverse event that is both serious and, in the opinion of the reporting Investigator, believed with reasonable probability to be due to one of the trial treatments, based on the information provided. |
| Suspected Unexpected Serious Adverse Reaction (SUSAR) | A serious adverse reaction, the nature and severity of which is not consistent with the information about the medicinal product in question set out:   - in the case of a product with a marketing authorisation, in the summary of product characteristics (SmPC) for that product - in the case of any other investigational medicinal product, in the investigator’s brochure (IB) relating to the trial in question. |

## 10.2 Causality

The relationship of each adverse event to the trial medication will be determined by a medically qualified individuals (DMSB) according to the following definitions:

**Related**: The adverse event follows a reasonable temporal sequence from trial medication administration. It cannot reasonably be attributed to any other cause.

**Not Related**: The adverse event is probably produced by the participant’s clinical state or by other modes of therapy administered to the participant.

## 10.3 Procedures for Recording Adverse Events

All AEs occurring during the trial / or until the last date of follow up of the last participant, that are observed by the Investigator or reported by the participant, will be recorded on the AER (annexure 4), whether or not attributed to trial medication. All adverse effects will be notified to ERC FMS, USJ and SCCOT of the National Medicinal Regulatory Authority of Sri Lanka as per time frames and guidelines specified by these bodies

The following information will be recorded: description, date of onset and end date, severity, assessment of relatedness to trial medication, other suspect drug or device and action taken. Follow-up information should be provided as necessary.

The severity of events will be assessed on the following scale: 1 = mild, 2 = moderate, 3 = severe. AEs considered related to the trial medication as judged by a medically qualified investigators or the Sponsor will be followed either until resolution, or the event is considered stable.

## 10.4 Reporting Procedures for Serious Adverse Events

All SAE will be reported to the ERC and the SCOCT within 24 hours from the time the study team, finds out.

## 10.5 SUSAR Reporting

All SUSARs will be reported by the PI to the ERC and to the SCOCT. For fatal and life-threatening SUSARS, this will be done no later than 7 calendar days after the investigators are first aware of the reaction. Any additional relevant information will be reported within 8 calendar days of the initial report. All other SUSARs will be reported within 15 calendar days.

## 10.6 Data Safety Monitoring Committee

This clinical trial will be monitored by a Data and Safety Monitoring Board (DSMB) which will comprise of the following members:

1. Dr. Panduka Karunanayake: Department of Medicine, University of Colombo

2. Prof. Priyadharshani Galappathy: Department of Pharmacology, University of Colombo

3. Dr. Carukshi Arambepola: Department of Community Medicine, University of Colombo

4. Dr. Preethi Wijegoonawardena: Consultant Family Physician

# STATISTICS

## Description of Statistical Methods

The interim analysis will be at half way when half the number of patients are recruited. Statistical significance will be calculated for the interim analysis using the

• χ² test for categorical variables in the two groups

• one-way analysis of variance for continuous variables in the two groups

• Kruskal–Wallis for non-parametric continuous variables in the two groups

• Fisher exact test for categorical variables with small sample size in the two groups ( example when the side effects are small in numbers)

• Intention to Treat (ITT) - ITT analysis will be included as this ignores noncompliance, protocol deviations, withdrawal, and anything that happens after randomization

At the end of the trial, the primary endpoint will be the reduction in the number of individuals who develop DHF. And the secondary efficacy endpoints included duration of reduction in stay at hospitals and side effects. Analysis of covariance (ANCOVA) for the primary endpoint and for secondary endpoints will be used as a statistical method in addition to the above basic statistical methods if applicable. This will be supplemented by a repeated measures analysis.

The effect size will also be computed. Standardized mean difference (SMD), which is used for continuous measures such as leakage, and the number needed to treat, which is used for binary outcomes such as side effects in both groups. The SMD will be computed as the difference between drug and placebo divided by their standard deviation:

SMD = (Drug Improvement - Placebo Improvement) / Standard Deviation

## The Number of Participants

This is planned to evaluate rupatidine drug compared to a placebo in reducing the proportion of patients who develop vascular leak (DHF). The response rate of the active control drug, rupatidine in reduction of fluid leakage by Ultra sound scan was found to be 42.8% (p1) and in the standard treatment was 23.5% (p2). The number of participants per group required to detect a difference in the two groups with a significance level of 5% and a power of 1 - β is 280.


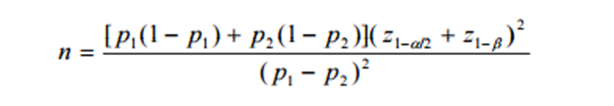


Given an anticipated dropout rate of 10% , the sample size needed per group is 280.

## The Level of Statistical Significance

The level of significance will be 0.05.

## Criteria for the Termination of the Trial

In the light of interim data the DSMC will inform the PI, if, in their view, there is proof beyond reasonable doubt that the data indicate that the trial should be terminated. A decision to inform the PI of such a finding will in part be based on statistical considerations and side effects. Appropriate proof beyond reasonable doubt cannot be specified precisely. A difference of at least 3 standard errors in the interim analysis of a major endpoint, which would be the number of patients going into DHF may be needed to justify halting or modifying the study prematurely.

## Procedure for Accounting for Missing, Unused, and Spurious Data.

Missing data will be described, for example, by presenting the number and percentage of individuals in the missing category. All data collected on data collection forms will be used, since only essential data items will be collected. No data will be considered spurious in the analysis since all data will be checked and cleaned before analysis.

## Inclusion in Analysis

All randomised participants, all dosed participants, all eligible participants will be included. If and when a patient is unblinded that patient will not be included in the analysis. Any data from a patient who is lost to follow up will be included until that point.

## Procedures for Reporting any Deviation(s) from the Original Statistical Plan

Deviations from the original statistical plan are unlikely. However, should they occur, then approval for such changes will be sought from the ERC and these changes will be reported in the final report and papers.

# DATA MANAGEMENT

## Source Data

Source documents are where data are first recorded, which the data collection form (Annexure 1). The data will be obtained by direct interview of the patient by one of the investigators and also by accessing the hospital clinical records and laboratory reports.

All documents will be stored safely in confidential conditions. On all trial-specific documents, other than the signed consent, the participant will be referred to by the trial participant number/code and not by name.

## Access to Data

Direct access will be granted to authorised representatives from the Sponsor, host institution and the regulatory authorities to permit trial-related monitoring, audits and inspections.

## Data Recording and Record Keeping

All trial data will be entered on to paper (data collection forms) and subsequently entered to data base in the main computer at the CDR. The participants will be identified by a unique trial specific number and/or code in any database. The name and any other identifying detail will NOT be included in any trial data electronic file.

# QUALITY ASSURANCE PROCEDURES

The trial will be conducted in accordance with the current approved protocol, GCP, relevant regulations and standard operating procedures.

Regular monitoring will be performed according to GCP. Data will be evaluated for compliance with the protocol and accuracy in relation to source documents. Following written standard operating procedures, the monitors will verify that the clinical trial is conducted and data are generated, documented and reported in compliance with the protocol, GCP and the applicable regulatory requirements.

The DSMB will meet once the trial has recruited half of the participants. The DSMB comprises of a physician, a clinical pharmacologist, a statistician, a general practitioner and a patient who is medically qualified.

# SERIOUS BREACHES

A serious breach is defined as “A breach of GCP or the trial protocol which is likely to affect to a significant degree –

(a) the safety or physical or mental integrity of the subjects of the trial; or

(b) the scientific value of the trial”.

In the event that we suspect a serious breach is suspected the Sponsor must be contacted within 1 working day. In collaboration with the C.I., the serious breach will be reviewed by the Sponsor and, if appropriate, the Sponsor will report it to the REC committee, Regulatory authority and the NHS host organisation within seven calendar days.

# ETHICAL AND REGULATORY CONSIDERATIONS

## Declaration of Helsinki

We will ensure that this trial is conducted in accordance with the principles of the Declaration of Helsinki. We will also ensure that this trial is conducted in accordance with relevant regulations and with Good Clinical Practice.

## Approvals

The protocol, informed consent form, participant information sheet and any proposed advertising material will be submitted to the Ethics Review Committee (ERC), and the SCOCT for written approval. We will submit and, where necessary, obtain approval from the above parties for all substantial amendments to the original approved documents.

## Reporting

We will submit yearly progress reports throughout the clinical trial to the ERC and an End of Trial notification and final report will be submitted to the ERC.

## Participant Confidentiality

The trial staff will ensure that the participants’ anonymity is maintained. The participants will be identified only by a participant ID number on all trial documents and any electronic database. All documents will be stored securely and only accessible by trial staff and authorised personnel. The trial will comply with the Data Protection Act, which requires data to be anonymised as soon as it is practical to do so.

## Expenses and Benefits

**Benefits for the patients for taking part in the study (immediate)**

- Results of NS1 antigen test and dengue antibodies will be made available to the patient and the treating physician. This would help in the management of the patient.
- As rupatadine was found to reduce the proportion of patient developing DHF and other complications in our previous trial, it could reduce the clinical disease severity of the patient and need for admission.

**Reimbursement of patients**

- All patients who are taking part in the study will be given Rs. 1000 as travel and inconvenience payments for taking part in this study. They will be further reimbursed with Rs. 500 when they come for the follow up visit on day 14 since onset of illness.

# FINANCE AND INSURANCE

Funding will be provided by the Centre for Dengue Research, University of Sri Jayewardenapura. This study or the participants have not been covered by any insurance scheme.

# PUBLICATION POLICY

The data of this trial will be presented at relevant meetings and will be published at reputed international journals. All data will be made available.

# REFERENCES

1. Bhatt S, Gething PW, Brady OJ, Messina JP, Farlow AW, Moyes CL, et al. The global distribution and burden of dengue. Nature. 2013;496(7446):504-7.

2. Mortality GBD, Causes of Death C. Global, regional, and national life expectancy, all-cause mortality, and cause-specific mortality for 249 causes of death, 1980-2015: a systematic analysis for the Global Burden of Disease Study 2015. Lancet. 2016;388(10053):1459-544.

3. Deen J. The Dengue Vaccine Dilemma: Balancing the Individual and Population Risks and Benefits. PLoS medicine. 2016;13(11):e1002182.

4. Martina BE, Koraka P, Osterhaus AD. Dengue virus pathogenesis: an integrated view. Clinical microbiology reviews. 2009;22(4):564-81.

5. Jeewandara C, Gomes L, Wickramasinghe N, Gutowska-Owsiak D, Waithe D, Paranavitane SA, et al. Platelet activating factor contributes to vascular leak in acute dengue infection. PLoS neglected tropical diseases. 2015;9(2):e0003459.

6. Nettis E, Delle Donne P, Di Leo E, Calogiuri GF, Ferrannini A, Vacca A. Rupatadine for the treatment of urticaria. Expert opinion on pharmacotherapy. 2013;14(13):1807-13.

7. Mullol J, Bousquet J, Bachert C, Canonica WG, Gimenez-Arnau A, Kowalski ML, et al. Rupatadine in allergic rhinitis and chronic urticaria. Allergy. 2008;63 Suppl 87:5-28.

8. Valero A, de la Torre F, Castillo JA, Rivas P, del Cuvillo A, Antepara I, et al. Safety of rupatadine administered over a period of 1 year in the treatment of persistent allergic rhinitis: a multicentre, open-label study in Spain. Drug safety : an international journal of medical toxicology and drug experience. 2009;32(1):33-42.

9. Katiyar S, Prakash S. Pharmacological profile, efficacy and safety of rupatadine in allergic rhinitis. Primary care respiratory journal : journal of the General Practice Airways Group. 2009;18(2):57-68.

10. Abajian M, Curto-Barredo L, Krause K, Santamaria E, Izquierdo I, Church MK, et al. Rupatadine 20 mg and 40 mg are Effective in Reducing the Symptoms of Chronic Cold Urticaria. Acta Derm Venereol. 2016;96(1):56-9.

11. Donado E, Izquierdo I, Perez I, Garcia O, Antonijoan RM, Gich I, et al. No cardiac effects of therapeutic and supratherapeutic doses of rupatadine: results from a 'thorough QT/QTc study' performed according to ICH guidelines. British journal of clinical pharmacology. 2010;69(4):401-10.

12. Church MK. Efficacy and tolerability of rupatadine at four times the recommended dose against histamine- and platelet-activating factor-induced flare responses and ex vivo platelet aggregation in healthy males. The British journal of dermatology. 2010;163(6):1330-2.

13. Garcia-Gea C, Ballester MR, Martinez J, Antonijoan RM, Donado E, Izquierdo I, et al. Rupatadine does not potentiate the CNS depressant effects of lorazepam: randomized, double-blind, crossover, repeated dose, placebo-controlled study. British journal of clinical pharmacology. 2010;69(6):663-74.

14. Shepard DS, Undurraga EA, Halasa YA, Stanaway JD. The global economic burden of dengue: a systematic analysis. The Lancet infectious diseases. 2016;16(8):935-41.

15. WHO, editor. Comprehensive guidelines for prevention and control of dengue fever and dengue haemorrhagic fever. SEARO, New Delhi, India: World Health Organization; 2011.

16. McDowell Torres D, Stevens RD, Gurakar A. Acute liver failure: a management challenge for the practicing gastroenterologist. Gastroenterology & hepatology. 2010;6(7):444-50.

17. Espie CA, Kyle SD, Hames P, Gardani M, Fleming L, Cape J. The Sleep Condition Indicator: a clinical screening tool to evaluate insomnia disorder. BMJ Open. 2014;4(3):e004183.

18. Seet RC, Quek AM, Lim EC. Post-infectious fatigue syndrome in dengue infection. J Clin Virol. 2007;38(1):1-6.

19. Garcia MN, Hause AM, Walker CM, Orange JS, Hasbun R, Murray KO. Evaluation of prolonged fatigue post-West Nile virus infection and association of fatigue with elevated antiviral and proinflammatory cytokines. Viral immunology. 2014;27(7):327-33.

20. Vaughn DW, Nisalak A, Solomon T, Kalayanarooj S, Nguyen MD, Kneen R, et al. Rapid serologic diagnosis of dengue virus infection using a commercial capture ELISA that distinguishes primary and secondary infections. The American journal of tropical medicine and hygiene. 1999;60(4):693-8.

21. Sang CT, Cuzzubbo AJ, Devine PL. Evaluation of a commercial capture enzyme-linked immunosorbent assay for detection of immunoglobulin M and G antibodies produced during dengue infection. Clinical and diagnostic laboratory immunology. 1998;5(1):7-10.

22. WHO. Dengue guidelines for diagnosis, prevention and control. New edition ed. Southeast Asian Office of the WHO World Health Organisation; 2009.

23. Levey AS, de Jong PE, Coresh J, El Nahas M, Astor BC, Matsushita K, et al. The definition, classification, and prognosis of chronic kidney disease: a KDIGO Controversies Conference report. Kidney international. 2011;80(1):17-28.
