# Supplementary material for: Efficacy of rupatadine in reducing the incidence of dengue haemorrhagic fever in patients with acute dengue: A randomised, double blind, placebo-controlled trial
Source: PLoS Negl Trop Dis. 2022 Jun 1;16(6):e0010123. doi: 10.1371/journal.pntd.0010123 (PMC9191706; doi:10.1371/journal.pntd.0010123)
Supplement: Attachment — Submitted filename: Answers to reviewer questions 04.02.2022.docx [file pntd.0010123.s003.docx]

4^th^ February 2022

## Prof. William B. Messer**,**

## **Associate Editor**,

PLOS Neglected Tropical Diseases

Dear Professor Messer,

**Submission of manuscript ‘Efficacy of rupatadine in reducing the incidence of dengue haemorrhagic fever in patients with acute dengue: a randomised, double blind, placebo-controlled trial’**

We wish to thank the reviewers for carefully going through our manuscript and the useful suggestions they have made. We have incorporated all these changes in the revised version and letter and have addressed all issues raised by the reviewers. We have highlighted the changes in the revised manuscript.

**Reviewer #1: The manuscript is generally well written and data has been clearly presented.**

**My comments are as follow:**

**1.      The abstract and summary are misleading in their claims. I would suggest the authors tone down the language used.**

**Line 35-36: Therefore, patients given rupatadine were 55% less likely to develop DHF than those on the placebo (relative risk 0.68, 95% CI 0.41 to 1.08). This statement is not accurate as the statistical analysis did not reach significance.**

**Response:** We thank the reviewer for this suggestion and we apologise if the language sounded misleading. We have rephrased the sentences and included that the results are not statistically significant in the revised version of the manuscript.

**Lines 63-65: Rupatadine was previously shown to reduce endothelial dysfunction in vitro, reduced vascular leak in dengue mouse models and to reduce the extent of pleural effusions and thrombocytopenia in patients with acute dengue. I would suggest that the authors clarify that the reduction in vascular leak observed in humans only showed a trend and was a post hoc analysis in the prev study published in Sci Report.**

**Response:** We thank the reviewer for this important question, and we have included that the reduction in the extent of the pleural effusions and reduction in thrombocytopenia was assessed in a post ad-hoc analysis. We have included this in the author summary in the revised version of the manuscript as suggested.

However, in our previous study in the post ad-hoc analysis, we did observe a significant difference in the extent of pleural effusion on day 8 and a significant difference in the platelet counts on day 7 since onset of illness. We did not observe a trend but statistically significant differences. A trend was only observed in an analysis carried out in patients who were recruited ≤3 days in our previous study, where we found that those who were given rupatadine were less likely to develop pleural effusions or ascites and had a significantly shorter duration of illness.

**Lines 70-71: Same comments as above.**

**Response:** We thank the reviewer for pointing this out. We have made the relevant changes as suggested by the reviewer.

**2.      It would be important to state clearly under the statistical analysis section whether the 140 per arm in the original sample size calculation included anticipated drop-outs. And with the final sample size of 123/126, what is the power of this trial to detect a difference in the reduction of fluid leakage in the 2 arms. If the current trial size is adequately powered to address the primary endpoint, the authors should not be advocating for yet another larger trial of rupatadine monotherapy in their summary and conclusion of the discussion. A larger trial is unlikely to be useful at the expense of cost and time if the current trial size is powered adequately.**

**Response:** We thank the reviewer for this question. The final sample size is under powered to detect a difference in the reduction of fluid leakage in the two arms. For instance, when looking at the primary end points - 9.7% patients developed DHF in the treatment arm compared to 22 (17.5%) in the placebo arm. This had a power effect of 43.3%, which implies the current sample size is inadequate. We agree with the reviewer that a larger trial is unlikely to be useful and based on the results of this trial and the previous trial, it does appear that rupatadine has some effect. However, based on the overall findings we believe that rupatadine alone is unlikely to significantly reduce the proportion of patients with acute dengue developing DHF, and therefore, is would not be useful to carry out a large expensive trial. Instead, it would be important to explore the usefulness of using rupatadine in combination with another drug, possibly montelukast. We have included this in the revised version of the manuscript.

**3.      It is interesting that patients in the rupatadine arm experienced less hepatic tenderness, vomiting and headache. These findings should be discussed further as to the possible/postulated mechanisms of actions involved. Liver inflammation is one of the causes of hepatic tenderness in acute dengue. How did the liver enzymes compare in the 2 arms at baseline?**

**Response:** We thank the reviewer for this question and the findings are very interesting. As we only obtained blood samples daily for the full blood count, liver enzymes were not monitored in any of the patients at the time of recruitment (at the outpatient department) or during the course of the study period. This was not planned as the hospital did not have the capacity to carry out liver function tests on a daily basis in all patients recruited to the study. We agree with the reviewer, it would have been important to monitor liver enzymes. We have mentioned this as a limitation and discussed the possibilities of reduction in liver tenderness and headache in those given rupatadine.

**4. In the discussion lines 314-316: Therefore, although we only 123 patients completed treatment in the rupatadine arm and 126 in the placebo arm, we found that those who were given rupatadine were twice as less likely (9.7%
compared to 17.5%) to develop DHF compared to those on the placebo arm.
This statement is misleading as the result was not statistically significant.**

**Response:** We thank the reviewer for this comment and as suggested by the reviewer, we have amended this sentence in the revised version of the manuscript.

**It is also noteworthy that the rupatadine arm had fewer patients experiencing platelet counts of less than 50,000 compared to placebo although this did not translate to fewer bleeding events overall.**

**Response:** We thank the reviewer for this important observation. We have included this in the discussion in the revised version as suggested by the reviewer.

**Reviewer #2: This manuscript by Malavige and colleagues report the findings of a phase 2 clinical trial into the rupatadine as a therapy to prevent dengue hemorrhagic fever (DHF). The authors have previously found that rupatadine reduced endothelial cell dysfunction and hence vascular leakage in experimental models. In a previously published proof-of-concept trial, they also showed that rupatadine reduced the rate of pleural effusion although treatment did not reduce the rate of patients that eventually developed DHF compared to placebo. In this trial, the goal was to test the hypothesis that rupatadine, when given early following illness onset, would reduce the extent of vascular leakage and hence the rate of DHF in dengue patients. The authors randomised 123 patients into receiving rupatadine and 126 patients into receiving placebo. The primary endpoint, reduction in relative risk of DHF, was not statistically significant. There were secondary endpoints that were statistically significant. The authors surmised that the primary endpoint was compromised by the failure to recruit 140 cases per arm due to the reorganization of the public health resource allocation to manage the covid-19 outbreak in Sri Lanka. They thus concluded that the findings support a larger trial to demonstrate the efficacy of rupatadine in preventing DHF.**

**This is a well written manuscript that reports an important clinical trial. Several areas, however, need attention. These are:**

1.      The description of the a priori sample size calculation in the main manuscript is inconsistent with the supplementary information. The main text omitted the mention of n=140 was derived to cater for the anticipated 10% dropout rate. The number of patients that need to be included for a statistically meaningful analysis, using the equation shown in the supplementary information, should thus be reported, in addition to the final estimate of n=140.

Response:

**2.      Following from the above comment, the inclusion of n=123 and n=126 in the active and placebo arms, respectively, is within the range of the a priori calculated sample size. The conclusion in lines 43-45 is thus not convincing. What is clear from this and the previous smaller clinical trial carried out by the authors is that data from the experimental models have exaggerated the therapeutic effect of rupatadine on vascular leakage. The assumed response rate is thus overly optimistic. The authors should thus consider a more circumspect discussion on the trial findings and what the data tells on the a priori assumptions. They should also discuss whether a response rate that is more reasonable and lower than presently assumed would still be clinically useful. A recommendation for future trials should only be made after a more thorough and circumspective discussion on the findings.**

**Response:** We thank the reviewer and completely agree with the points made. We do think that rupatadine might have some effect, but unlikely to be useful by itself. The in vitro and animal therapeutic effects observed were at very high concentrations of rupatadine. In our trial dose (the accepted higher limit of the rupatadine dose in humans), did not achieve concentrations anywhere near the ones that were used in our in vitro data and possibly the mouse studies. We have included these details in the discussion of the revised version of the manuscript.

**3.      The authors should also consider taking the opportunity to discuss biological insights that can be gleaned from their trials. Clearly, rupatadine does have some effect on reducing liver dysfunction in dengue patients, as well as reduce the rate/intensity of some of the classical dengue symptoms. What can the dengue field glean about dengue pathogenesis from these different outcomes?**

**Response:** We thank the reviewer for this suggestion. Rupatadine does seem to have some effect on liver dysfunction and possibly reducing vascular leak. We think it would be important to investigate the mechanisms by which rupatadine might be reducing liver dysfunction and also having some effect on vascular leak. We have included these points in the discussion of the revised version of the manuscript.

**4.      Lines 110-118. This justification for anti-DHF therapy is unnecessary. Dengue is both a medical and a global health problem. The need for such therapy is independent of the problems caused by covid-19.**

**Response:** We agree with the reviewer that justification for anti-DHF therapy in unnecessary. However, we believe that it is more urgent for development of anti-dengue therapeutics because of COVID-19. For instance, in Sri Lanka, we are experiencing a large dengue outbreak since early 2020 along with a large COVID-19 outbreak due to Omicron. Because both infections have a similar presentation (fever, headache, malaise, myaligia and fatigue), many dengue patients are not diagnosed until they present with complications, or in DHF and some have co-infections. This new scenario does complicate things so much in dengue endemic countries. Therefore, development of drugs for dengue was important, but now it is urgent.

**5.      Line 150. Please specify if the NS1 antigen test used was point-of-care test or ELISA.**

**Response**: We thank the reviewer for this question. We recruited the patients at the outpatients department, if they tested positive from a point of care NS1 antigen test. We have included this in the revised version of the manuscript.

**6.      Table 2. It is unclear if the platelet counts shown in the table represent the nadir of thrombocytopenia. Please clarify.**

**Response:** We apologize for not mentioning this. We have included that the platelet counts that are shown in table 2 are in fact the nadir.

**7.      Figure 2A is somewhat concerning. The platelet count in the treated arm continued on the downward trend even at the end of the monitoring period whereas those in the placebo arm has shown an upswing. Could rupatadine be delaying the platelet nadir or even prolong thrombocytopenia?**

**Response:** We thank the reviewer for this question. Figure 2A is somewhat misleading. This is because at day 9, only a few patients who were still in hospital were included in the analysis. This included only 6 patients on rupatadine and 7 patients on the placebo. As this data is misleading and only represents a very low sample number, we have removed the data point shown on day 9.

Thank you for considering our manuscript for publication with PLOS Neglected Tropical Diseases.

Yours Sincerely,

Prof. Neelika Malavige
