# Supplementary material for: Efficacy of rupatadine in reducing the incidence of dengue haemorrhagic fever in patients with acute dengue: A randomised, double blind, placebo-controlled trial
Source: PLoS Negl Trop Dis. 2022 Jun 1;16(6):e0010123. doi: 10.1371/journal.pntd.0010123 (PMC9191706; doi:10.1371/journal.pntd.0010123)
Supplement: Attachment — Submitted filename: Answers to reviewer questions 13.04.2022.docx [file pntd.0010123.s004.docx]

13^th^ April 2022

## Prof. William B. Messer**,**

## **Associate Editor**,

PLOS Neglected Tropical Diseases

Dear Professor Messer,

**Submission of manuscript ‘Efficacy of rupatadine in reducing the incidence of dengue haemorrhagic fever in patients with acute dengue: a randomised, double blind, placebo-controlled trial’**

We wish to thank the reviewers for carefully going through our manuscript and the useful suggestions they have made. We have incorporated all these changes in the revised version and letter and have addressed all issues raised by the reviewers. We have highlighted the changes in the revised manuscript.

We have also added the trial data that was used to generate figures and tables as a supplementary data.

**Reviewer #2: I thank the authors for addressing some of the concerns raised on their original submission. However, several issues remain to be fully resolved. These are:**

**1. The authors missed out on responding to my original comment #1. The number of patients that need to be included for a statistically meaningful analysis, based on the equation shown in the supplementary information and before accounting for anticipated dropout rates need to be reported. Without this important detail, the amended lines 215-217 remains unsubstantiated.**

**Response**: We thank the reviewer for the additional comments and apologise for not being clear regarding the sample size calculations. Due to the early termination of the trial because of unavoidable circumstances, this resulted in a 0.05 type I error. Therefore, the study was found to be underpowered with only 123 recruited to the treatment arm and 126 recruited to the placebo arm. Therefore, the post hoc power was found to be 43.3%, which was therefore, underpowered to evaluate the primary end point, which was the reduction in the proportion of patients developing DHF. We have mentioned this in the revised version of the manuscript.

**2. The removal of day 9 platelet count data from Figure 2A is incorrect. I fully understand the authors’ explanation, but removal of that data is not the way to solution. The authors should instead reinstate this data but provide an explanation in the figure legend on how the day 9 platelet count data should be interpreted.**

Response: We thank the reviewer for this comment. We have now reinstated the data on figure 2A and provided reasons for the observation in the figure legend.

**3. The amended Figure 2A without a corresponding change in Table 2 raises another concern: Was day 9 platelet count included in the analysis on the nadir of platelet count? Clearly, for some patients in the active arm, the platelet count may still be on the declining trend at day 9. The possibility that rupatadine treatment delays platelet recovery must be considered and instead of being brushed aside.**

**Response**: We thank the reviewer for this very important question. Of the 6 patients on rupatadine at day 9, two of them did have the lowest counts on day 9. For the analysis in table 2, their platelet counts on day 9 were taken as their lowest platelet count for the analysis.

In addition, we repeated all the analysis to find any further errors and we have made changes to the results for the duration of illness for those in the rupatadine arm. We apologize for the error in our previous data and regarding the duration of illness, which we have corrected and it is now significantly different between the treatment arm and the placebo arm.

**Minor comments:**

**1. Lines 35-37 is repetitive. Details on relative risk etc can be incorporated into the preceding sentence and the sentence in lines 35-37 can be deleted.**

Response: We thank the reviewer for this suggestion, and we have deleted this sentence in the revised version.

**2. Line 65. Post-ad hoc should be changed to post hoc.**

Response: We thank the reviewer for this suggestion, and we have deleted this word in the revised version.

**3. Lines 313-316. Please revise this sentence.**

Response: We thank the reviewer for this comment and we have rephrased this sentence.

**4. Depending on the sample size calculation and platelet count analysis, the conclusion may need to be phrased differently.**

Response: We have rephrased the conclusion as suggested by the reviewer in the revised version.

Thank you for considering our manuscript for publication with PLOS Neglected Tropical Diseases.

Yours Sincerely,

Prof. Neelika Malavige
