# Supplementary material for: Efficacy of rupatadine in reducing the incidence of dengue haemorrhagic fever in patients with acute dengue: A randomised, double blind, placebo-controlled trial
Source: PLoS Negl Trop Dis. 2022 Jun 1;16(6):e0010123. doi: 10.1371/journal.pntd.0010123 (PMC9191706; doi:10.1371/journal.pntd.0010123)
Supplement: Attachment — Submitted filename: Answers to reviewer questions 09.05.2022.docx [file pntd.0010123.s005.docx]

9^th^ May 2022

## Prof. William B. Messer**,**

## **Associate Editor**,

PLOS Neglected Tropical Diseases

Dear Professor Messer,

**Submission of manuscript ‘Efficacy of rupatadine in reducing the incidence of dengue haemorrhagic fever in patients with acute dengue: a randomised, double blind, placebo-controlled trial’**

We wish to thank the reviewers for carefully going through our manuscript and the useful suggestions they have made. We have incorporated all these changes in the revised version and letter and have addressed all issues raised by the reviewers. We have highlighted the changes in the revised manuscript.

**Reviewer 2's concern that the data in figure 2A suggest that rupatadine may delay recovery of platelet counts has not been fully addressed. As I read the reviewer's comments, it is clear that the possibility that rupatadine could delay platelet count recovery needs to be mentioned in the manuscript. The fact that platelet counts were not tracked to recovery means that any claims of rupatadine treatment diminishes thrombocytopenia are not fully substantiated by the data. Please add the observation that neither arm demonstrated a recovery in platelet counts by day 9 to the results section and then add to the discussion an acknowledgement that platelet data must be interpreted carefully as neither arm showed recovery of platelet count by end of data collection.**

Response: We thank the reviewer for this comment. We agree that while rupatadine appears to reduce the extent of the thrombocytopenia, some individuals did have quite significant thrombocytopenia and there seems to be a delay in recover in these individuals. We have mentioned this in the results and also in the discussion. On day 9 of illness, 6 patients on rupatadine and 7 patients on the placebo were still being followed up and therefore, were included in the analysis. Of the 6 patients on rupatadine 1/6 had a platelet count of <10,000 cells/mm3 on day 9, and 2/6 patients had platelet counts between 50,000 to 100,000 cells/mm3. In the placebo arm 4/7 patients had platelet counts between 50,000 to 100,000 cells/mm3 on day 9 of illness. We believe that the platelet count of 8000 cells/mm3 of one patient on rupatadine would have skewed the data to look that those on rupatadine have a delayed recovery. However, we have not mentioned this in the revised version and have mentioned what was recommended by the reviewer.

**Additional minor comments**

**line 190 - Ultra sound should be one work and not capitalized**

**Lines 353-360 can be deleted as they are redundant with early text.**

Response: We thank the reviewer for these comments. We have amended these in the revised version as suggested.

Thank you for considering our manuscript for publication with PLOS Neglected Tropical Diseases.

Yours Sincerely,

Prof. Neelika Malavige
